# HCoVDB: a comprehensive database encompassing viral genomes, drug targets, and therapeutics of human coronaviruses

**DOI:** 10.1093/database/baaf079

**Published:** 2025-11-26

**Authors:** Pan Zhang, Tianxiang Ouyang, Xiaowen Hu, Jie Huang, Biao Xiao, Zhijian Huang, Xingyang Shi, Xinyi Wu, Linying Chen, Yongkang Wu, Hanyue Wang, Ying Zhang, Guangdi Li, Hui Liu, Lei Deng

**Affiliations:** Infection Control Center, Xiangya Hospital of Central South University, No. 87 Xiangya Road, Kaifu District, Changsha 410008, Hunan, China; National Clinical Research Center for Geriatric Disorders, Xiangya Hospital of Central South University, No. 87 Xiangya Road, Kaifu District, Changsha 410008, Hunan, China; School of Computer Science and Engineering, Central South University, No. 932 South Lushan Road, Yuelu District, Changsha 410075, Hunan, China; School of Computer Science and Engineering, Central South University, No. 932 South Lushan Road, Yuelu District, Changsha 410075, Hunan, China; Hunan Provincial Key Laboratory of Clinical Epidemiology, Xiangya School of Public Health, Central South University, No. 172 Tongzipo Road, Yuelu District, Changsha 410013, Hunan, China; Hunan Provincial Key Laboratory of Clinical Epidemiology, Xiangya School of Public Health, Central South University, No. 172 Tongzipo Road, Yuelu District, Changsha 410013, Hunan, China; School of Computer Science and Engineering, Central South University, No. 932 South Lushan Road, Yuelu District, Changsha 410075, Hunan, China; School of Computer Science and Engineering, Central South University, No. 932 South Lushan Road, Yuelu District, Changsha 410075, Hunan, China; School of Computer Science and Engineering, Central South University, No. 932 South Lushan Road, Yuelu District, Changsha 410075, Hunan, China; School of Computer Science and Engineering, Central South University, No. 932 South Lushan Road, Yuelu District, Changsha 410075, Hunan, China; School of Computer Science and Engineering, Central South University, No. 932 South Lushan Road, Yuelu District, Changsha 410075, Hunan, China; School of Computer Science and Engineering, Central South University, No. 932 South Lushan Road, Yuelu District, Changsha 410075, Hunan, China; School of Computer Science and Engineering, Central South University, No. 932 South Lushan Road, Yuelu District, Changsha 410075, Hunan, China; Hunan Provincial Key Laboratory of Clinical Epidemiology, Xiangya School of Public Health, Central South University, No. 172 Tongzipo Road, Yuelu District, Changsha 410013, Hunan, China; College of Computer and Information Engineering, Nanjing Tech University, No. 30 Puzhu South Road, Jiangbei New District, Nanjing 211816, Jiangsu, China; School of Computer Science and Engineering, Central South University, No. 932 South Lushan Road, Yuelu District, Changsha 410075, Hunan, China

## Abstract

Over the past few decades, coronavirus outbreaks have been reported globally. To date, seven human coronaviruses have been identified, among which only SARS-CoV-2 has been extensively studied, resulting in the development of several approved antiviral drugs. To effectively combat both current and emerging coronaviruses, there is an urgent need for a comprehensive database that consolidates information on all known human coronaviruses and their potential antiviral compounds. In response, we present HCoVDB—a comprehensive database that integrates genomic data, viral proteins, and antiviral agents with demonstrated *in vitro* or *in vivo* activity against the seven human coronaviruses. Compared to existing coronavirus databases, HCoVDB offers three distinctive features: (i) a curated collection and annotation of over 4 million genomic sequences from all seven human coronaviruses, including key amino acid substitutions that influence viral fitness, drug resistance, and immune evasion; (ii) a protein–drug docking platform for predicting the binding interactions of antiviral agents with demonstrated activity; and (iii) an extensive compilation of antiviral agents, along with their chemical properties and antiviral efficacy profiles (IC_50_, EC_50_, or CC_50_) as reported in the literature. Overall, HCoVDB provides a valuable resource for tracking the evolutionary dynamics of coronaviruses and accelerating the development of broad-spectrum antiviral agents against coronavirus infections in the future.

**Database URL**: http://hcovdb.denglab.org/

## Introduction

Coronaviruses (CoVs) are enveloped, positive-sense, single-stranded RNA viruses that belong to the subfamily Orthocoronavirinae within the family Coronaviridae, a monophyletic lineage classified under the order Nidovirales [1]. Since 1965, seven distinct human coronaviruses have been identified, including HCoV-229E [2], HCoV-OC43 [3], SARS-CoV [4], HCoV-NL63 [5], HCoV-HKU1 [6], MERS-CoV [7], and SARS-CoV-2 [8]. Among these, HCoV-NL63 and HCoV-229E are classified as α-coronavirus genus, while the remaining five are β-coronaviruses genus. It is known that over the past two decades, three highly pathogenic coronaviruses (SARS-CoV-2, SARS-CoV, and MERS-CoV), all of which have been classified as β-coronaviruses, have emerged and led to a high risk of mortality in humans [9]. Of note, different human coronaviruses cause various levels of risk from mild cold-like symptoms to severe symptoms such as acute respiratory distress syndrome, cytokine storms, multi-organ failure, and even death [10–13]. The coronavirus pandemic has underscored the pressing demand for the development of broad-spectrum antiviral drugs to control both the currently prevalent coronavirus variants and potential new coronaviruses that may emerge in the future [14].

Genomic surveillance plays an important role in tracking viral evolutionary trajectory [15, 16]. A significant fraction of the sequences are mainly catalogued by two repositories, GenBank [17] and GISAID [18]. GenBank (https://www.ncbi.nlm.nih.gov/genbank/) provides an open-access annotated collection of nucleotide sequences and their protein translations from all known viruses. In contrast, GISAID (https://gisaid.org/) mainly focuses on influenza and coronaviruses, with millions of genomic sequence records that contribute to a better understanding of viral evolution. Genetic alterations of coronavirus sequences could potentially alter the structure of the proteins targeted by antiviral drugs, thereby undermining the efficacy of drugs designed to bind to those proteins [19, 20]. From another perspective, the administration of antiviral drugs can impose selective pressure on the virus, driving the emergence of drug-resistant mutations that allow the virus to circumvent the drug efficacy, mostly among those immunocompromised patients who undertake long-term antiviral treatment [21–23]. Therefore, to elucidate the association between genomic alterations of coronaviruses and antiviral drug resistance, we conducted a comprehensive functional annotation of mutation sites within the genomes or amino acid substitutions of seven coronaviruses in our database.

The lifecycle of coronaviruses encompasses several critical processes, including fusion with host cells, the release of the viral genome, protein translation and synthesis, genomic replication and assembly, packaging, and subsequent release [24]. These stages require interactions between viral RNA and host proteins, as well as interactions between viral proteins and host proteins [25–27]. Currently, the development of antiviral agents that target coronavirus proteins or essential host proteins has become a primary therapeutic strategy for combating coronavirus infections. For the treatment or the prophylaxis of SARS-CoV-2, small-molecule agents (e.g. nirmatrelvir/ritonavir, molnupiravir, and remdesivir) and various monoclonal antibodies have been marketed, whereas no antiviral agent has been approved for the treatment of the other six human coronaviruses [28]. For example, nirmatrelvir is a peptidomimetic inhibitor of the SARS-CoV-2 main protease (also known as 3C-like protease, 3CLpro), and its binding site has been elucidated by X-ray crystallography [25]. However, recent studies suggest that some amino acid substitutions within drug-binding pockets of SARS-CoV-2 proteins can confer resistance to approved anti-SARS-CoV-2 agents. For instance, a study identified 22 naturally occurring mutations located in the binding pocket of nirmatrelvir, and these variants exhibited resistance to nirmatrelvir [19]. Moreover, the emerging variants also drive the interests of developing broad-spectrum agents with potent activities against SARS-CoV-2 variants. To elucidate the possible interplay between drug resistance and variants of human coronaviruses, it is worth reporting a comprehensive information on drug resistance mutations within the full-length genome of seven human coronaviruses.

Several platforms have been established with curated resources related to the genomics and evolution of the coronavirus. For instance, CoVdb compiled the coronavirus genome sequences and offers online analyses of its evolutionary trajectory [29]. However, this tool did not address the relationship between the viral evolution and the efficacy of antiviral drugs. CovInter summarized the interactions between human coronavirus RNA and host proteins, providing insights into the biological functions of host proteins [25]. However, CovInter has not compiled therapeutic targets and small-molecule compounds that can block the function of these targets, thus its practical clinical value is limited. The COVID-19 pandemic has led to a surge in studies aimed at unravelling the evolutionary trajectories of coronaviruses and the comprehensive impact of specific mutation sites on virus replication, transmissibility, immune evasion, and drug sensitivity. For instance, the D614G mutation site has been widely observed in Variants of Concern (VOCs), with numerous studies suggesting that this mutation significantly enhances viral infectivity and adaptability. However, a database that summarizes all amino acid mutations and their impact on antiviral agents is still lacking.

In this study, we propose a comprehensive database, HCoVDB, which integrates the genomic sequences, drug–target interactions, and chemical information of antiviral agents for seven human coronaviruses. First, we integrated and annotated the genomic mutations through an exhaustive review of the biomedical literature, as well as identified significant mutation sites and their impacts on therapeutic efficacy. We also collect small-molecule agents and essential drug targets, and develop a functional module to visualize the protein–drug molecular docking. Essential details regarding antiviral drugs and drug combinations are also incorporated into the database. HCoVDB seeks to offer a mechanistic interpretation of the inherent relationship between small-molecule compounds and mutation patterns of coronaviruses at both the genomic and protein levels. We believe this resource can clarify the relationship between viral genome evolution and drug efficacy, thus promoting the development of broad-spectrum antiviral agents.

## Materials and methods

### Data sources

We obtained genomic sequence data for seven human coronaviruses from the National Center for Biotechnology Information Virus Module (NCBI Virus; https://www.ncbi.nlm.nih.gov/labs/virus/vssi/), a dedicated community for the storage and analysis of all known virus data. The selected coronaviruses include SARS-CoV-2, SARS-CoV, MERS-CoV, HCoV-HKU1, HCoV-NL63, HCoV-OC43, and HCoV-229E. This data underwent standardized annotation and statistical analysis. To ensure the integrity and accuracy of the data, we applied two filtering criteria based on host species and nucleotide completeness, ultimately retaining only complete genomic sequences with humans as the host.

To clarify the relationship between viral genetic alterations and the resistance to antiviral agents, we manually compiled functional annotations of mutation sites in coronavirus strains from established databases, including GenBank and GISAID, as well as articles sourced from PubMed and Google Scholar. Our initial step involved a thorough collection of mutation sites through genomic mutation analysis, followed by using the nomenclature of each mutation site to identify relevant studies that confirmed their functionality. We also used functional keywords such as geographical distribution, clinical information, immune evasion, and drug resistance in relation to mutation sites to refine our literature search.

**Figure 1.**
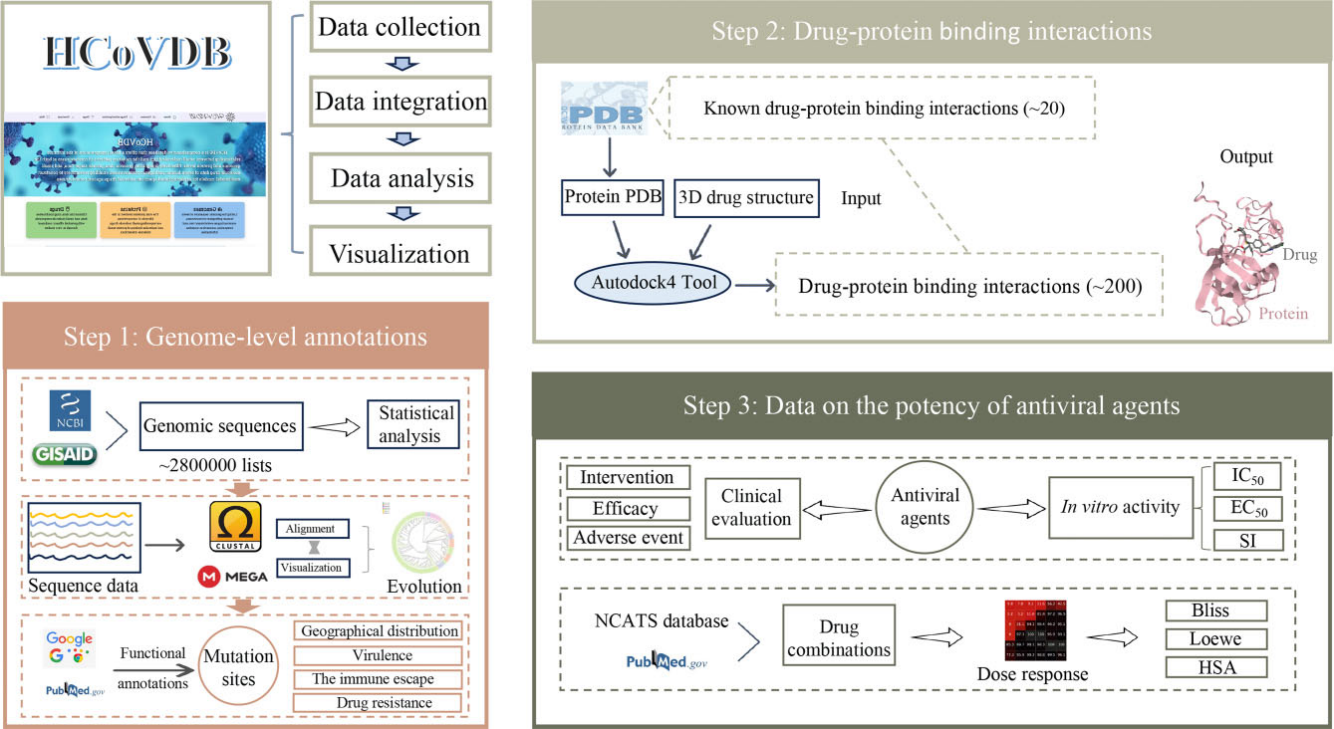
The framework of the HCoVDB database. This database comprises three core components: (1) genomic-level annotations, (2) target protein–compound interactions, and (3) antiviral activity data of small molecules.

Figure 1 visualizes our workflow for manual curation and the integration of biological data resources. Pharmaceutical agents, including investigational drugs, clinical trial agents, and drug combinations, were compiled from various sources, including the PubMed database, Google Scholar, ClinicalTrials.gov, and NCATS. We conducted a thorough manual curation of all publications related to small molecules with potential antiviral activity against coronaviruses, supported by *in vitro* or *in vivo* experiments. Specifically, we employed keywords associated with human coronaviruses, such as SARS-CoV-2, SARS-CoV, and MERS, in conjunction with terms like drug, inhibitor, and IC_50_ in our search queries. This effort resulted in a total of 1702 distinct publications. Table 1 presents the number of publications corresponding to each coronavirus. Of note, small molecules predicted by *in silico* methods were excluded from our inclusion criteria.

**Table 1.** Statistics on manually curated data for drugs with potential anti-coronavirus activity *in vitro* and clinical trials

		Investigative compounds			
	Years of occurrence	Retrieved publications	Number of drugs	Evaluation metrics	Clinical trials
SARS-CoV-2	2019	1291	548	IC50, EC50, CC50	1162
MERS-CoV	2012	154	65	IC50, EC50, CC50	9
HCoV-HKU1	2005	5	5	IC50, EC50	–
HCoV-NL63	2004	17	8	IC50, EC50, CC50	–
SARS-CoV	2003	93	82	IC50, EC50, CC50	26
HCoV-OC43	1967	52	8	IC50, EC50, CC50	–
HCoV-229E	1965	90	25	IC50, EC50, CC50	–

### Sequence alignment

To investigate the evolutionary relationships among seven human coronaviruses, we performed multiple sequence alignment of genomic sequences using MAFFT V7 [30], a cutting-edge tool designed for rapid sequence alignment that guarantees the reliability and accuracy of the results. Additionally, we constructed an evolutionary tree using IQ-TREE [31]. Finally, we integrated this evolutionary tree into HCoVDB and enabled interactive visualization through D3.js.

### Molecular docking

For small molecules that have been experimentally validated to inhibit viral proteins, we gathered 3D structures in SDF format for all drugs from PubChem and obtained protein PDB structures from the PDB database. Subsequently, we conducted molecular docking between proteins and small-molecule drugs using AutoDock Vina, except for those complexes that were already resolved through X-ray crystallography. AutoDock Vina generated different binding poses for each protein–small-molecule pair based on various models. We selected and presented the result with the highest binding energy.

### Database development

The collective data resource is stored and managed using MySQL database. The backend infrastructure is implemented using the Spring framework. The frontend user interface is constructed with Vue.js. D3.js, a highly versatile data visualization library, is used to render complex data structures into insightful visuals. It is worth mentioning that the 3D viewer tool for visualizing molecular structures is implemented using JSmol, a feature-rich molecular visualization library known for its ability to provide interactive and dynamic representations of complex molecular structures.

## Results

### Genomic sequences

**Figure 2.**
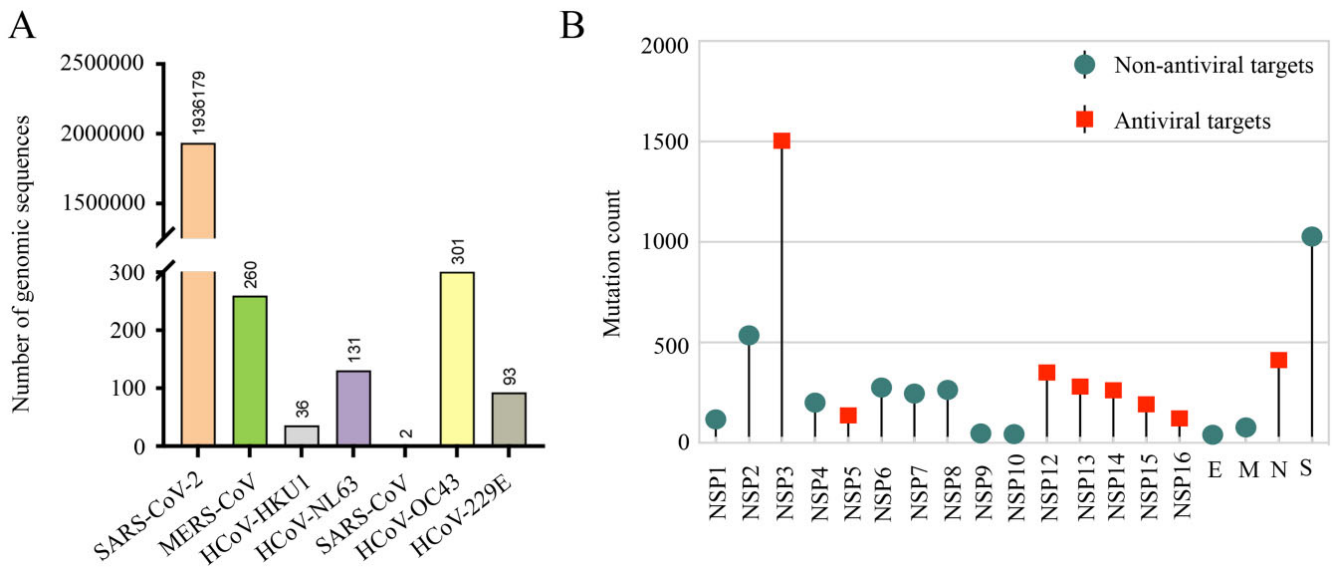
Summary of genome sequences and mutations in human coronaviruses. (A) Number of genome sequences available for seven human coronaviruses. (B) Summary of mutation counts in the SARS-CoV-2 genome.

The HCoVDB database houses a total of ∼2.0 million complete genome sequences representing all seven human-infecting coronaviruses. Figure 2A presents the detailed numbers of genomic sequences collected for each of the seven human coronaviruses. Users can obtain detailed sequence information by entering a provided ID number into the web browser interface. Furthermore, a sampling method is utilized to select hundreds of records from these seven coronaviruses to construct the evolutionary tree. This tree offers a rich visual representation of genomic relationships among various human coronaviruses. Each branch of the tree represents a specific coronavirus genome, enabling users to access detailed information related to that particular virus. This feature illustrates the intricate genomic and evolutionary relationships among viral lineages.

### Amino acid mutations

The distribution of 6141 amino acid mutations within the SARS-CoV-2 genome is illustrated in Fig. 2B. To provide informative data regarding these mutations, we collect annotations of each substitution site reported in scientific literature, thereby establishing the ‘viral mutations’ module. This module contains various metadata, such as organism, lineage, mutations, gene, and protein. Through manual review of the publications, we summarize a description field that indicates the effect of mutation site on drug efficacy or clinical outcome. This is represented by a series of labels, each denoting a specific aspect: ‘D’ for Drug Sensitivity, ‘R’ for Drug Resistance, ‘G’ for Geographical Distribution, ‘V’ for Virulence, ‘I’ for Immune Escape, ‘T’ for Transmission, and ‘C’ for Clinical Outcome.

**Figure 3.**
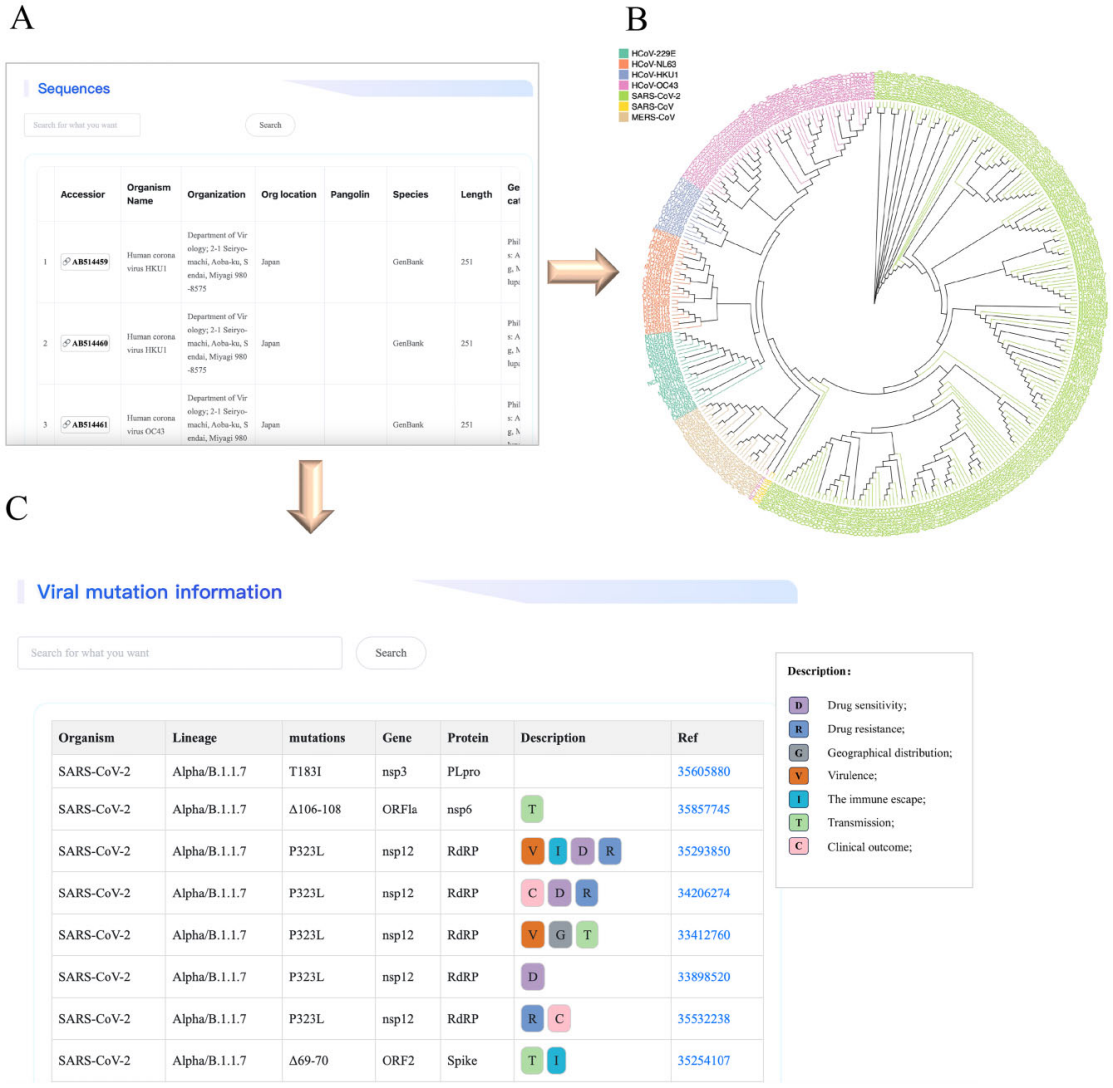
Screenshot illustrating an example of genome exploration in the HCoVDB database. (A) A detailed list of sources and basic information on genomic sequences from human coronaviruses. (B) An evolutionary tree constructed from representative genomic sequences. (C) A functional annotation of viral mutation sites.

Users could access the detailed data by searching the name or other related keywords of mutation sites. For instance, when a user is interested in the P323L mutation of SARS-CoV-2, he can input a keyword such as ‘P323L’ or ‘RdRP’ to launch the search; this will bring him to browse detailed information about this mutation site, including therapeutic drugs, drug resistance, immune escape, and geographical distribution, etc. This module helps users to explore the evolutionary patterns of amino acid mutations in human coronaviruses, as well as their impact on viral virulence and immune escape. Furthermore, identification of key amino acid mutations near the drug-binding pockets will contribute to the optimization of antiviral drugs for the inhibition of viral variants. Figure 3 provides an overview of the three web interfaces related to genomic data, encompassing genome sequence information, the phylogenetic tree, and genomic mutation annotations.

### Drug–target interactions

**Figure 4.**
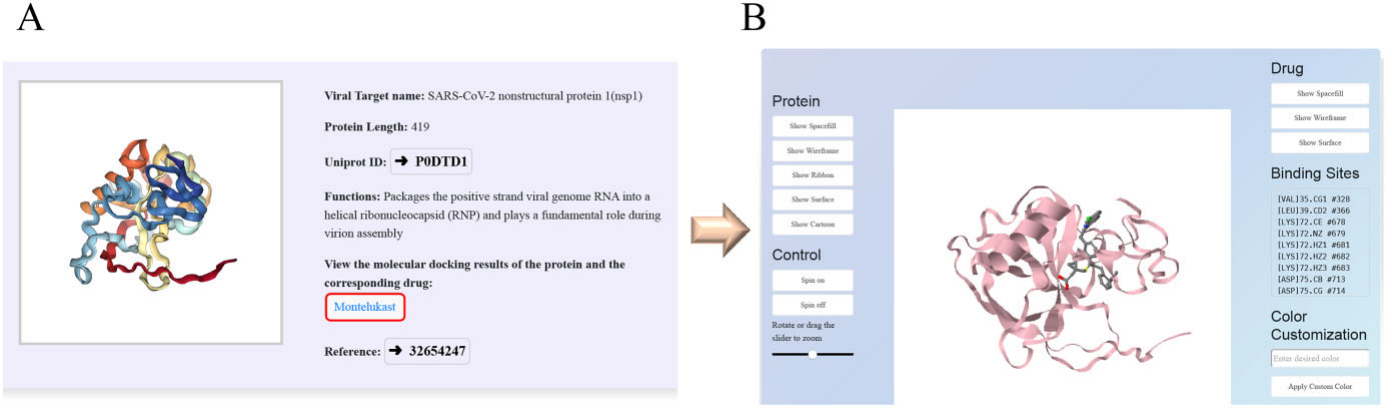
Screenshot illustrating an example of target exploration in the HCoVDB database. (A) Basic information on the SARS-CoV-2 protein NSP1. (B) Options to view detailed molecular docking results by selecting the drug ‘Montelukast’.

Our database includes drug targets derived from both viral and host proteins. These targets have been confirmed through *in vitro* experiments to be susceptible to specific inhibitors and to possess potential antiviral properties. Users can input a protein name into the search box to obtain information about the corresponding target. Clicking on a drug name enables users to view the online molecular docking results between the targets and the drugs. Within the docking panel, users can utilize buttons on the left or right to adjust the display modes for either proteins or drugs. Furthermore, users have the option to modify the display settings for protein–drug interactions and the binding pocket sites of proteins. Figure 4 illustrates a case study featuring the drug Montelukast and the target protein SARS-CoV-2 NSP1, along with a 3D structural visualization of the molecular docking results.

### Therapeutic drugs

A growing number of drug screening studies and clinical trials are dedicated to discovering new compounds or repurposing existing approved drugs. HCoVDB has gathered information on 1200 drugs currently in clinical trials and 700 compounds under investigation, as outlined in the ‘Drugs’ module. Our website enables users to efficiently search for and browse critical information related to these clinical trials, including trial phases, participant counts, disease conditions, study durations, and primary outcomes. Notably, users can click on the ‘register number’ link to be directed to the clinicaltrials.gov website for additional information.

The pharmaceutical agents under investigation are derived from confirmed compounds through *in vitro* experiments, rather than computational predictions. The information encompasses drug targets, such as viral proteins and human host proteins, as well as drug sensitivity metrics such as IC_50_, CC_50_, or EC_50_ values. The HCoVDB database primarily concentrates on crucial targeting sites such as the receptor-binding domain (RBD), papain-like protein, and receptor-binding motif (RBM). Furthermore, this module contains links to the PDB database, facilitating a three-dimensional visualization of the interactions between drug molecules and target proteins. By clicking on the PMID links, users can consult the publications that reported the therapeutic drug against coronaviruses. Users are granted unrestricted access to download the dataset from our website. We are confident that these data resource would expedite the evaluation of the potential drugs, thereby providing valuable suggestion to the research community for the development of drugs against human coronaviruses. Additionally, our HCoVDB database provides clinicians with the latest progress on antiviral discovery, which contribute to their better management of personalized treatment against human coronaviruses.

## Conclusion

To our knowledge, HCoVDB is the first comprehensive database that combines human coronavirus genomes, antiviral drug-binding sites within both viral and host proteins, and extensive information on antiviral agents with demonstrated clinical and *in vitro* activities against human coronaviruses. HCoVDB has been functioning reliably for several months, with ongoing enhancements to its interface and performance. Users can safely access and download all relevant data without requiring a login. Although the WHO declared an end to COVID-19 as a global health emergency in May 2023, SARS-CoV-2 variants are still circulating globally and the need for broad-spectrum agents against human coronaviruses still persists [32]. The emergence of viral variants and potential drug resistance mechanisms present significant challenges to effectively treating human coronaviruses on a global scale. Given the enduring and unpredictable threat posed by human coronaviruses, we believe that HCoVDB provides a crucial data resource for the discovery of broad-spectrum antiviral drugs.

## Data Availability

HCoVDB is a publicly and freely accessible database that is updated regularly. All data used in this manuscript can be accessed at http://hcovdb.denglab.org/.
